# Wanted Dead or Alive: A Correlate of Protection Against Dengue Virus

**DOI:** 10.3389/fimmu.2019.02946

**Published:** 2019-12-16

**Authors:** Lázaro Gil, Alejandro Martín, Laura Lazo

**Affiliations:** Center for Genetic Engineering and Biotechnology, Havana, Cuba

**Keywords:** dengue virus, vaccines, antibodies, cell-mediate immunity, protection, antibody-dependent enhancement of infection

The licensing of Dengvaxia^®^ 4 years ago seemed to signal an end to the dry spell that has plagued the development of dengue vaccines for almost a century. Regrettably, Dengvaxia^®^ turned out to partially mimic primary infection in dengue-naïve individuals, thus increasing the risk of severe dengue upon subsequent infections in a scenario not dissimilar to that occurring during natural secondary infections (1). On the flip side, Dengvaxia^®^'s failure has contributed to our understanding of what could constitute a protective immune response in the context of dengue, especially regarding the role of neutralizing antibodies, although it has also tempered expectations on the future outcome of ongoing dengue vaccine efficacy trials. Developing dengue vaccines is far from a trivial endeavor, due to the lack of suitable animal models, the need for a tetravalent formulation effective against all four viral serotypes -and the associated problem of waning immunity, which may render a vaccine recipient susceptible to severe dengue during natural infection- and, especially, due to the fact that a reliable correlate for protection has not been found. Until the latter hurdle is cleared, efficacy trials of dengue vaccines will need to be conducted based on clinical endpoints, following virologically-confirmed dengue cases of any severity due to any serotype (2).

Dengue is a mosquito-borne viral infection that affects tropical and subtropical areas of the world (3, 4). It is caused by four antigenically related but distinct dengue virus (DENV) serotypes belonging to the family *Flaviviridae*, genus *flavivirus* (5). These viruses produce around 390 million infections and 20,000 deaths annually worldwide (6). DENV are transmitted mainly by mosquitoes from *Aedes* genus. The infection results in different clinical outcomes: asymptomatic (most common) or mildly symptomatic illness, uncomplicated dengue fever, or more severe disease including plasma leakage, hemorrhage, and vascular collapse (1, 7).

A lot of studies have supported the protective role of the humoral immune response, specifically, neutralizing antibodies. The first evidence of the protective role of antibodies was described by Blanc and Caminopetros in 1929, when healthy volunteers were inoculated with sera from DENV-infected individuals and 10 days later were challenged with a wild-type strain of DENV (8), observing partial protection.

As early as 1969, Halstead and colleagues described, in our opinion, the most important epidemiological observation related with DENV infection (9). Babies born from DENV-immune mothers were protected during the first 3–4 months of their life and this protective response was undoubtedly mediated by neutralizing antibodies transferred from mothers to children. However, when the levels of neutralizing antibodies decreased due to the catabolism, children became susceptible to develop the severe form of the disease during the first natural infection, even with the same virus serotype that previously infected their mothers. Similar observations published by Kliks et al. (10), supported the previous one. These and other studies constituted the bases to propose the occurrence of a key phenomenon associated to the immunopathogenesis of DENV infection: the antibody-dependent enhancement (ADE) of infection. Several studies conducted *in vitro* and *in vivo* sustain the ADE phenomenon and its implication during DENV infection and vaccination.

Several observations of the immunopathogenesis of DENV infection and the contribution of antibodies to this phenomenon have been exemplified by many authors (11–14). One of them was the experiment reported by Halstead and coworkers in 1979 using rhesus monkeys. Animals were inoculated with human cord blood serum from DENV-immune or non-immune individuals and later infected with DENV-2. As a result, all animals receiving DENV-immune sera showed an increased viremia in comparison with that detected in animals inoculated with DENV-negative sera (15).

Obviously, this phenomenon has important implications for vaccine developers. Nevertheless, all vaccine candidates developed up to date have as their main goal, the elicitation of neutralizing antibodies. The protective role of this type of antibodies is undoubtedly, but their protective capacity is limited by their affinity and most important by their concentration, which decreases with the time.

The potential risk of ADE is the main challenge associated with the development of a safe vaccine against DENV (16). A vaccine inducing sub-protective anti-DENV antibodies may be inefficient, and also may cause ADE-mediated severe disease. In addition, despite the induction of a protective antibody response, its levels could wane and get concentration after vaccination that can mediate the ADE of the infection (14, 17, 18). The measurement of neutralizing antibodies *in vitro* may not accurately correlate with protection *in vivo*, as it was demonstrated by results of the phase IIb clinical trial performed with Dengvaxia^®^ (19). Dengvaxia^®^ partially mimics primary infection and increases the risk of severe dengue during subsequent infection, similar to that observed during a natural secondary dengue infection (20). One of the most accepted hypotheses to explain the non-outstanding results obtained with Dengvaxia^®^ is the absence of T-cell epitopes in the chimeric viruses and the occurrence of ADE of the infection (21).

Unlike Dengvaxia^®^, the National Institute of Health of US has developed a live-attenuated tetravalent vaccine candidate; using molecular attenuation and chimerization strategies (22). These viruses contain the capsid and non-structural proteins from three out of four DENV, which are the main targets of cytotoxic and antiviral cytokine-secreting CD4^+^ and CD8^+^ T cells (23, 24). The cellular immune response generated by this vaccine candidate is comparable to those observed after natural dengue infection: a broad response to structural and non-structural proteins after monovalent vaccination and a T-cell response against highly conserved epitopes from non-structural proteins after tetravalent immunization (25). Additionally, this vaccine candidate is able to protect humans after challenge with partial attenuated strains of DENV-2 or DENV-3 (26).

In the last years the protective role of the cell-mediate immune response against DENV has taken a crucial role. Several mice experiments have demonstrated that CD8^+^ and even CD4^+^ T cells contribute to protection, controlling the viral disease or reducing viral load in blood and different organs (27–29). Nevertheless, some researchers have associated this response with the development of severe dengue during heterologous infection (30, 31), a phenomenon known as original antigenic sin. This hypothesis postulates that during secondary infection, the expansion of pre-existing lower avidity cross-reactive memory T cells dominates the response over that of naïve T cells which have higher avidity for the new DENV serotype. It is further hypothesized that peptide variants derived from the second serotype (known as altered peptide ligands) can induce a response that is qualitatively different from that induced by the original antigen, producing a different pattern of cytokines (32). These altered T-cell responses may produce a “cytokine storm” during heterologous secondary infection and thus contribute to the immunopathogenesis of severe dengue disease (33). In 2006, Mongkolsapaya and colleague demonstrated this phenomenon, but only using lymphocytes from one individual (30), which limits, in our opinion, its generalization. Four years later, the same group tried to support their hypothesis analyzing a higher number of individuals who experienced primary or secondary infections. The results demonstrated the predominance of a functional cross-reactive T cells response during the secondary heterologous infection, which showed cytotoxic activity, avoiding the development of severe dengue (31) (please, see figure 3 of this paper).

The association of the original antigenic sin with the T-cell response is contradictory; because the heterologous T-cell response not always produces severe disease in infants. As it was previously mentioned, severe dengue in infants, from endemic areas, generally occurs between 6 and 12 months after delivery (34), despite the fact that they have never been infected with DENV and lack of DENV-specific memory T cells (35). Besides, an important study has shown that the CD8^+^ T-cell response and the capillary leakage do not match in time, suggesting that CD8^+^ T cells are not responsible for severe signs in children infected with DENV (36).

T cells may contribute to protection against homologous or heterologous DENV infection. Some studies suggest the occurrence of homologous reinfection, specifically with a different genotype of the same virus (37–39), but this infection is in the majority of cases, asymptomatic. We hypothesize that despite the role of the ADE phenomenon that always occurs during a secondary infection, the memory T-cell response generated during the primary infection is able to control the homologous secondary infection by a different virus genotype. To support our hypothesis, we can analyze recent results published by Juraska and coworkers (40). These authors observed a genotype-mediated protection induced by Dengvaxia^®^ against DENV-4. This vaccine only protected individuals against the natural infection with same virus genotype that was used to obtain the chimeric yellow fever-dengue-4 virus. Nevertheless, this result is completely expected taking into account that Dengvaxia^®^ does not induce DENV-specific T-cell responses (41) that can control the replication of any genotypes. Although, genotype-specific antibodies could be considered a correlate of protection, in practice the development of vaccine candidates inducing this kind of response is actually difficult. In that case, seasonal vaccines must be obtained, depending on the circulating genotypes, but for DENV this is impossible. That is why, genotype-specific antibodies must not be considered as a correlate of protection.

DENV-specific human CD4^+^ T and CD8^+^ T cells proliferate, produce IFNγ, and lyse infected cells in humans who suffer a primary infection (42, 43). In fact, high frequencies of DENV-specific IFNγ-producing T cells are present in children with subclinical infections, in comparison with children with symptomatic secondary DENV infection (44, 45).

A deep study conducted in 2013 demonstrated the correlation between a polyfunctional CD8^+^ T-cell response and protection against the disease (23). This study proposes that the HLA haplotype defines the quality of the cellular immune response. There are certain alleles that during secondary infection inadequately stimulate the CD8^+^ T cells that show a dysfunctional immune response (46). Therefore, in the context of ADE, during heterologous virus infections, a dysfunctional T-cell response cannot control the viral load and infected individual can develop severe dengue. This hypothesis could explain epidemiological data showing that only 3–5% of secondary infections develop the severe form of the disease, despite the existence of cross-reactive antibodies with the potential capacity to induce ADE of infection. On the contrary, remaining secondary infections do not develop alarm signs, neither severe dengue. With a very high probability, in this group of individuals, the memory T-cell response generated during the primary infection limits the viral replication, even in the context of ADE of infection, avoiding complications of the disease. Although, results obtained in animal models could not translate to humans, several studies conducted in mice during the last years have demonstrated the protective role of cross-reactive T cells and its capacity to control the disease in presence of sub-protective antibodies (47, 48).

An additional evidence to support the potential role of T cells as a correlate of protection against DENV could be the findings described by Weiskopf et al. (49). These researchers highlight that during DENV infection there are differences in the immune responses depending on the infecting serotype. These differences have important implications for vaccine design and development. The majority of the DENV-2-specific T cell responses in human are directed against non-structural proteins (23), whereas about one-third of the DENV-3-specific response is directed against the membrane and envelope proteins (49). This issue could explain the low protective efficacy of Dengvaxia^®^ against DENV-2 and the higher protective rate observed against DENV-3 (19). Despite the presence of neutralizing antibodies against both serotypes, the protective efficacy against these viruses could be related with the unequal antigen-induced T-cell responses.

To our knowledge, only one vaccine candidate has been designed to induce only a cell-mediated immunity (CMI). This vaccine candidate is based on the recombinant capsid proteins of DENV expressed in the bacteria *Escherichia coli* and these proteins form nucleocapsid-like particles (NLP) after their incubation with a synthetic oligonucleotide, containing CpG motives to stimulate the immune response. NLP from DENV-2 elicited in mice and monkeys an IFNγ-secreting cell response with cytotoxic activity that successfully reduced the viral load after a homologous viral challenge (50, 51). In the year 2016, the same authors demonstrated that the tetravalent formulation of NLP elicited a cellular immune response that significantly reduces viral load in mice after challenge with each DENV and the viremia in monkeys after challenge with DENV-3 (52). It is important to highlight, that anti-capsid antibodies do not play any role in the protective capacity of this vaccine candidate. Anti-capsid antibodies do not recognize the virus neither neutralize the infection (50, 53). In accordance with these results, Weiskopf and coworkers demonstrated that the capsid proteins of DENV are the main target of cytolytic and IFNγ-producing CD4^+^ T cells generated in humans during a natural infection (24, 54). This last finding supports the use of capsid proteins as a vaccine candidate.

The tetravalent formulation of NLP could reduce viral load modulating the clinical course of the disease, from severe dengue to mild dengue fever or from mild dengue fever to asymptomatic infection, thus preventing the appearance of severe signs of the disease. This CMI-based vaccine candidate has as the main advantage the absence of virus-binding antibodies, avoiding the ADE phenomenon. Therefore, the risk to develop a pathogenic response will be very low even if the antigen-induced immunity could not be as high as expected (55).

However, in the year 2014, Slifka published a review with the main aim to define a potential correlate of protection against DENV (56). The review analyzed the results published by Monath et al. (57) and Guirakhoo et al. (58), from clinical studies conducted to determine the role of pre-existing Yellow fever virus (YFV)-specific immunity in the replication of attenuated viruses based on the strain 17D of YFV. The authors evaluated the vaccine strain YFV-17D and chimeras, in which the envelope and PrM proteins of YFV-17D were changed by the envelope and PrM proteins of DENV-2 (58) or Japanese encephalitis virus (JEV) (57). The chimeric viruses, YFV-DENV-2 and YFV-JEV, have the non-structural proteins of YFV-17D and in consequence the same CD4^+^ and CD8^+^ T-cell epitopes. However, these chimeras cannot be neutralized by YFV-17D-specific antibodies. This scenario provided the opportunity to measure the role of T cells in the absence of neutralizing antibodies. The vaccination of naïve individuals with the three viruses (YFV-17D, YFV-DENV-2, or YFV-JEV) produced a detectable viremia in more than 80% of individuals. However, when YFV-17D-vaccinated individuals were inoculated with each virus, no viremia was observed after the inoculation with YFV-17D. On the contrary, the chimeric viruses (YFV-DENV-2 or YFV-JEV) produced viremias similar to those observed in YFV-17D-naïve subjects.

Taking into account these results, Slifka suggested that CD8^+^ and CD4^+^ memory T-cell responses against YFV, in absence of neutralizing antibodies, do not reduce viral load after flaviviruses infection. In our opinion, these results only demonstrate that the T-cell response does not control the viral load produced by YFV. Indeed, it has been demonstrated that protection induced by YFV-17D is mainly mediated by antibodies (59). A recent study conducted in mice to elucidate the immune mechanisms that underlie 17D-based vaccine efficacy demonstrated that antibodies and CD4^+^ T cells, but not CD8^+^ T cells contribute to protection after viral challenge (60). Therefore, we consider that the conclusion arose by Slifka is a complete misunderstanding of the results obtained in the clinical trials. The extension of his conclusion to all flaviviruses is incorrect because it was only demonstrated for YFV.

On the other hand, properly designed protection experiments in monkeys may provide data quite relevant to humans. A recent study reported by Borges and colleagues support that the evaluation of vaccine candidates in non-human primates that measure viremia and RNAemia after viral challenge and also determine cytokines associated with the severe form of the disease could be crucial to translate the results from animals to humans (61).

We recognize the protective role of neutralizing antibodies against DENV, but their protection capacity is limited as it has been demonstrated for several researchers during more than 60 years. Neutralizing antibodies are correlates of protection for other flavivirus, like YFV or JEV, but unfortunately not for DENV. Dengue is an acute disease and ancient dogmas state that in this kind of infection, antibodies are the main arm to confer protection. However, DENV infection up-regulates the expression of MHC-I molecules, a common mechanism of many flaviviruses to avoid the action of the innate immune system (62). This mechanism facilitates the virus to reach the viral load necessary to be transmitted to a mosquito, guarantying its persistence in the nature. However, the up-regulation of the expression of MHC-I molecules constitutes another evidence supporting the protective role of the CMI.

The scientific community has not accepted T cells response as a feasible correlate of protection, despite all the experimental evidences accumulated during the last 10 years supporting that. T cells can only reduce viral load, but in DENV infection this is very important, due to viremia correlates with the severity of the disease. Finally, we think that a vaccine against DENV must not induce virus-binding antibodies due to their demonstrated risk. Vaccines based on the induction of cellular immune response could be the potential solution against this expanding and threatening human pathogen and T-cell response can be the so prized correlate of protection. Why not?

## Author Contributions

LG wrote and reviewed the manuscript. AM reviewed the manuscript. LL wrote and reviewed one part of the manuscript.

### Conflict of Interest

The authors declare that the research was conducted in the absence of any commercial or financial relationships that could be construed as a potential conflict of interest.
